# Intracranial Mycotic Aneurysm in a Left Ventricular Assist Device Patient: A Complication to Avoid

**DOI:** 10.7759/cureus.12401

**Published:** 2020-12-31

**Authors:** Pavida Pachariyanon, Arunee T Motes, Nandini Nair

**Affiliations:** 1 Internal Medicine, Texas Tech University Health Sciences Center, Lubbock, USA; 2 Cardiology, Texas Tech University Health Sciences Center, Lubbock, USA

**Keywords:** lvad, mycotic aneurysm, cellular immunity, intracranial infectious aneurysm

## Abstract

In the current era of the increasing use of left ventricular assist devices (LVADs) as a bridge to transplant or destination therapy, early diagnosis and therapy of complications are imperative to provide a better quality of life and improve outcomes. This case illustrates how superficial infections can lead to drastic complications in the setting of LVADs. The lack of signs and symptoms of systemic inflammatory response could be explained by cellular immunity impairment in patients on LVAD support. The formation of aneurysms is enhanced in the LVAD population due to altered hemodynamic physiology. It is possible that the combination of impaired cellular immunity and altered hemodynamics of the present-day continuous flow LVADS increases the risk of mycotic aneurysm formation and rupture in patients infected with less virulent organisms.

## Introduction

Left ventricular assist devices (LVADs) have been increasingly used as a bridge to transplant and for destination therapy in end-stage heart failure patients. The event rate of LVAD-related infection in the first year after implantation remains high, approximately 30% to 40% [1] LVAD-related infections are also seen with the increasing use of third-generation LVADs [2]. We present here a rare case of a ruptured intracranial mycotic aneurysm secondary to Staphylococcus epidermidis (S. epidermidis) bacteremia in the setting of a superficial driveline infection.

## Case presentation

A 53-year-old African American male with non-ischemic cardiomyopathy underwent Heartmate II LVAD (Abbott Laboratories, Chicago, Illinois) implantation as destination therapy. He presented with intermittent yellowish discharge around the driveline exit site approximately 140 days post-implantation. The patient was afebrile. The physical examination showed mild redness, sloughed skin, and gelatinous discharge around the driveline exit site. The physical examination was otherwise unremarkable. Laboratory studies showed a normal white cell count of 6500/mm^3^. Multiple blood cultures grew oxacillin-resistant S. epidermidis from 140 days onwards for the next 60 days. He was treated with local wound care and sequentially with clindamycin, amoxicillin-clavulanic acid, and doxycycline orally depending on the susceptibilities. However, the patient continued to have multiple episodes of breakthrough oxacillin-resistant S. epidermidis bacteremia despite the resolution of the wound around the driveline exit site. Intravenous vancomycin was initiated. Computed tomography (CT) of the abdomen and chest showed no fluid collection around the LVAD or driveline. The trans-esophageal echocardiogram was unremarkable. Intravenous vancomycin was switched to daptomycin due to difficulty in assessing accurate levels.

**Figure 1:**
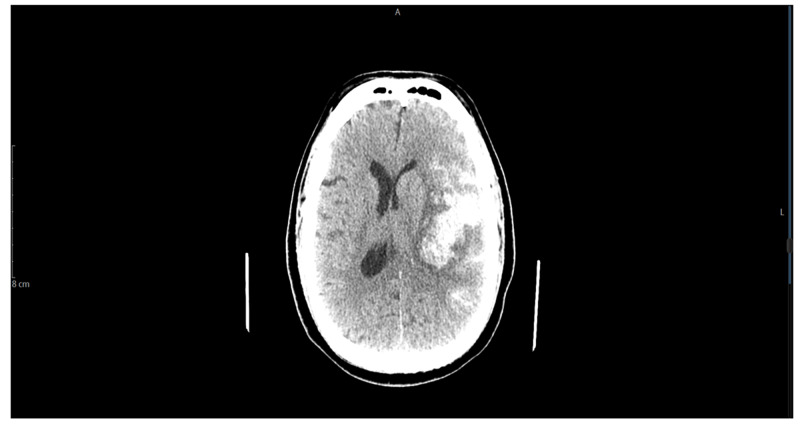
Non-contrast CT of the head showed a large intraparenchymal hemorrhage in the left external capsule, peri-insular region with subarachnoid hemorrhage CT: computed tomography

**Figure 2:**
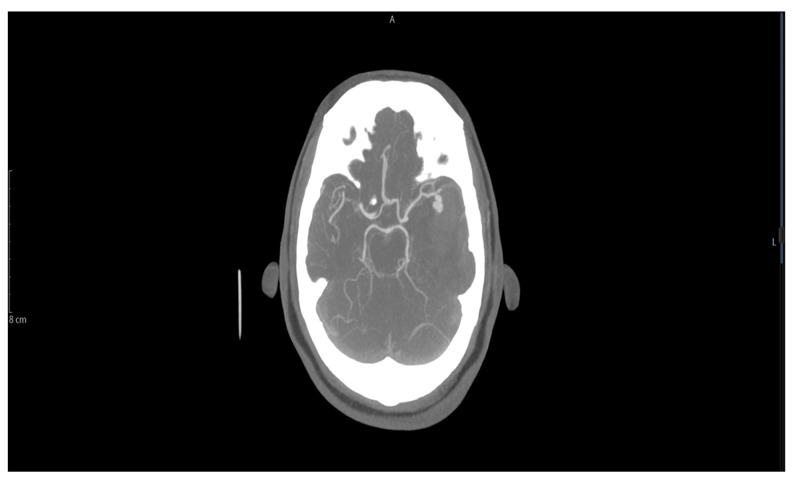
CT angiography of the head and neck showed a 10.7 x 9.6 x 7.6 mm bilobed-shaped fusiform aneurysm arising from the distal aspect of the posterior trunk of the left middle cerebral artery CT: computed tomography

Approximately two weeks after the intravenous antibiotic was initiated, the patient experienced sudden onset severe headache, profuse sweating, acute right hemiparesis, and rapidly became unresponsive. Non-contrast CT of the head showed large intraparenchymal hemorrhage in the left external capsule, a peri-insular region with subarachnoid hemorrhage, causing subfalcine herniation 10 mm toward the right (Figure 1). CT angiography of the head and neck showed a 10.7 x 9.6 x 7.6 mm bilobed-shaped fusiform aneurysm arising from the distal aspect of the posterior trunk of the left middle cerebral artery (Figure 2). His international normalized ratio was 1.25 while partial thromboplastin activation time was 34 seconds at the time. The patient underwent emergent left craniectomy with hematoma evacuation and aneurysm clipping. The patient’s neurologic status continued to deteriorate until his demise.

## Discussion

Approximately 40% of patients develop an infection after an LVAD implant with a higher incidence occurring early after implantation [1]. The LVAD-related infection has been associated with stroke, predominantly hemorrhagic stroke, and decreased one-year survival in this population [1-2]. Intracranial hemorrhage secondary to systemic infection could be a consequence of hemorrhagic transformation of an ischemic stroke, primary hemorrhage, and subarachnoid hemorrhage from septic emboli, vascular friability, or ruptured mycotic aneurysm as in infective endocarditis patients. Non-physiologic continuous-flow LVADs and the need for anticoagulation increase the risk of intracranial hemorrhage in the LVAD population [3].

Infectious intracranial aneurysm (IIA) refers to an aneurysm arising secondary to the inflammatory reaction caused by the microbial infection, predominantly bacterial. IIA is rare, only comprising 0.7%-5.4% of all intracranial aneurysms and usually associated with infective endocarditis [4]. It is interesting that S. epidermidis being less virulent still causes mycotic aneurysm formation in LVAD patients [5-11]. In this case, the patient did not present with signs or symptoms of systemic inflammatory response syndrome (SIRS). In another study, less than half of LVAD patients with bloodstream infection (BSI) met SIRS criteria [12]. The lack of signs and symptoms of SIRS, in this case, may be explained by cellular immunity impairment in the LVAD population [13-14]. It has been noted that Staphylococcus species and fungus are most commonly seen after LVAD implantation, suggesting that cellular immunity is impaired among LVAD recipients [15].

Driveline infections can be difficult to distinguish from driveline site trauma, which may lead to inadequate treatment, causing endovascular seeding of microorganisms from biofilm formation, thereby forming a focus for persistent bacteremia. Treatment of LVAD -related bacteremia typically includes four to six weeks of parenteral antibiotics, followed by long-term suppressive therapy with oral antibiotics and surgical debridement. Currently, there are no established guidelines for the surveillance of IIAs in active BSI without neurological manifestations in the LVAD population. This case illustrates that IIA surveillance and early diagnosis in asymptomatic LVAD patients could prevent fatality in this population. An abnormal state of monocyte and T-cell activation results in increased susceptibility of circulating CD4 T cells to activated cell death. Such abnormal immune activation leads to defects in cellular immunity resulting in a serious infection. The increased T-cell activation and selective loss of Th1 cytokine-producing CD4 T cells drive LVAD patients to develop B-cell hyperreactivity and poorly regulated immunoglobulin syntheses due to unopposed production of Th2 cytokines and increased Ligand-CD40 interactions [14]. It is postulated that immune dysregulation and function is a result of foreign biomaterial associated T-cell activation. This can only be prevented if the biomaterials are manufactured with altered physical properties or pharmacological inhibition of T cell response.

## Conclusions

Ruptured IIA is associated with high mortality. Early recognition and aggressive intervention of asymptomatic IIA in LVAD patients with active BSI may prevent the morbidity and mortality associated with aneurysm rupture. Further guidelines to actively monitor intracranial aneurysms in continuous-flow LVAD patients are warranted. Further investigation of immune dysregulation in LVAD recipients would pave the path to a better understanding of susceptibility to infections in this population.
